# Counteracting Physical Inactivity during the COVID-19 Pandemic: Evidence-Based Recommendations for Home-Based Exercise

**DOI:** 10.3390/ijerph17113909

**Published:** 2020-06-01

**Authors:** Fabian Schwendinger, Elena Pocecco

**Affiliations:** 1 Department of Circulation and Medical Imaging, Faculty of Medicine and Health Sciences, Norwegian University of Science and Technology, Prinsesse Kristinas gt. 3, 7006 Trondheim, Norway; 2 Department of Sports Science, Medical Section, University of Innsbruck, Fürstenweg 185, 6020 Innsbruck, Austria

**Keywords:** high intensity intermittent exercise, HIIT, physical inactivity, cardiorespiratory fitness, muscle strength, coronavirus

## Abstract

To reduce transmission of the coronavirus, from its initial outbreak in 2019 up to now, various safety measures have been enacted worldwide by the authorities that have likely led to reduced physical activity levels in the general population. This short communication aims to briefly outline the deteriorative consequences of physical inactivity on parameters of physical fitness and ultimately to highlight associated increases of cardiovascular disease risk and mortality. Finally, evidence-based practical recommendations for exercise that can be performed at home are introduced, to help avoid physical inactivity and therefore maintain or achieve good physical health.

## 1. Introduction

The coronavirus disease (COVID-19) has become a major health problem and has spread all over the world. Many countries have enacted quarantine or recommended people to stay at home and practice social/physical distancing. While these are important measures to reduce the spread of the disease, the amount of physical activity throughout the day may have been reduced considerably in the general population. This is apparent from data of the U.S. general population, showing a 48% reduction in 7 day rolling step count between 1 March and 6 April 2020 [1].

To date, there is strong evidence linking physical inactivity and sedentary behaviour to cardiovascular health, with low physical activity levels being associated with inferior health [2]. Furthermore, physical inactivity has been shown to account for 6–10% of the prevalence of various hypokinetic diseases, including cardiovascular diseases, diabetes mellitus type 2, as well as breast and colon cancer [2]. Therefore, the dramatic reductions in physical activity caused by the enacted safety measures may have serious consequences for public health in the long-run.

Thus, we aimed to briefly highlight the adverse physiological changes associated with physical inactivity, in order to clarify the impact of inactivity on cardiovascular disease risk, as well as mortality, and finally to introduce evidence-based exercise recommendations that are feasible for the general population in the current situation of the COVID-19 pandemic.

## 2. Impact of Physical Inactivity on Parameters of Physical Fitness

Several bed-rest studies have shown that a lack of physical activity is linked to the deterioration of both cardiorespiratory fitness (maximal oxygen uptake [V̇O_2max_]) and muscular capacity, e.g., muscle volume and maximal strength [3,4]. In the absence of physical activity, V̇O_2max_ drops gradually by about 0.3–0.4%/day [4]. Already after two weeks of bed rest, marked decreases in V̇O_2max_ and muscle volume are evident, with reductions by 7–15% (0.5–1.1%/day) and 6–8% (0.4–0.6%/day), respectively [3]. This worsening of physical fitness is accompanied by reduced contractile function of the muscle, as is apparent from decreased muscular strength and power [3]. Of note, there are differences in the duration and type of the enacted restrictions between countries, e.g., primarily social distancing in Norway or trust-based recommendations in Sweden vs. strict, long-term quarantine for >4 weeks in Italy and France. Considering the linear progression in the decline of V̇O_2max_ as a function of bed rest duration for at least 90 days, as shown in the literature [4], countries with longer restrictions may present greater deteriorations in the population’s health. Even though the actual situation caused by the COVID-19 pandemic may not cause the severe reductions seen in bed-rest studies, meaningful deteriorations in both cardiorespiratory fitness and muscular capacity can be expected and should, therefore, be prevented.

The levels of cardiorespiratory fitness and muscular strength are inversely associated with the incidence of cardiovascular disease (CVD) and all-cause mortality [5,6]. Indeed, a reduction of 1 MET (1 MET = 3.5 mL of oxygen·kg^−1^·min^−1^ in an average adult person at rest) in V̇O_2max_ is associated with an 18% increase in CVD incidence and a 15% reduction in survival [5]. It seems important to have at least moderate levels of these parameters to lower the all-cause mortality risk [6]. The following scenario demonstrates the consequences of reduced physical activity during the COVID-19 pandemic. A 55-year-old male subject with a V̇O_2max_ of 35.0 mL·kg^−1^·min^−1^ leads a sedentary lifestyle for four weeks; we assume his V̇O_2max_ would decrease only moderately, by 0.2% per day [4]. This would result in a 0.6 MET decrease after four weeks, which corresponds to 10.8% and 9.0% increases in CVD risk and all-cause mortality, respectively [5]. This example shows that such deteriorations are clinically relevant and should, therefore, receive particular attention. Fortunately, physical inactivity and the subsequent worsening of the aforementioned health parameters can easily be counteracted by physical activity and structured exercise [5,6].

## 3. Evidence-Based Exercise Recommendations

As there is a dose–response relationship between physical activity and major health outcomes, simply increasing the daily step count has a positive impact on mortality and CVD risk [7]. For individuals at home, one way to achieve this is to break sitting time with two minutes of walking every 20–30 min [8]. Taking the stairs whenever possible or adding a short walk to the daily schedule are other beneficial measures that should be implemented. Even 15 min per day of such moderate physical activities have been shown to contribute to reducing all-cause mortality [2]. Thus, every active minute counts.

However, to maximize health gains, we recommend structured exercise. Physical training should target both the cardiorespiratory system and the skeletal musculature to improve physical fitness and health [5,6]. Due to the enacted regulations, the majority of the general population has only limited space and equipment available to exercise. Hence, physical training should involve bodyweight exercises.

High-intensity interval training (HIIT), generally performed at 85–95% of maximum heart rate (HR_max_), is a more effective and time-efficient method in improving V̇O_2max_ than low- to moderate-intensity training [9]. Intermittent HIIT (I-HIIT) is a special form of HIIT, characterized by short intervals (typically 20 s) of near-maximal intensity and complete interruption of the exercise during breaks [10]. The short interval duration makes this training regime feasible for home-based exercise with limited space. The efficacy of I-HIIT in combination with bodyweight exercises has recently been confirmed in a randomized controlled trial with significant improvements in V̇O_2max_ (+1.4 METs, 11%) seen after four weeks [11]. Indeed, the training method adopted in this study allowed the subjects to reach exercise intensities that were >85% of HR_max_ [11].

To evoke improvements in muscle hypertrophy and strength, a wide range of resistance training intensities can be adopted by both untrained and trained adults, presupposing that the training is carried out with a high level of effort [12,13]. These findings support the use of bodyweight exercises. Yet, it should be noted that training with higher loads leads to greater improvements in one-repetition maximum [12,13]. 

**Figure 1:**
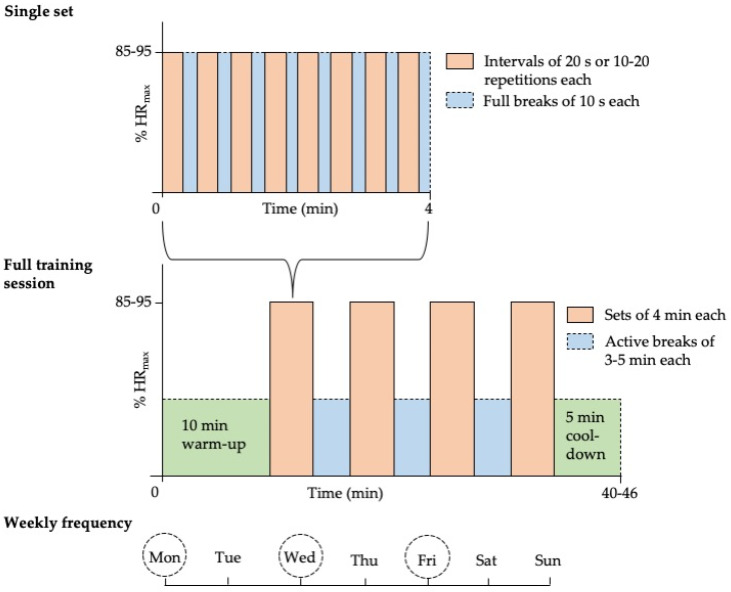
Evidence-based exercise recommendations. Intermittent high-intensity interval training (I-HIIT) should be performed as 3–4 sets, each consisting of 8 × 20 s intervals with 10 s of rest between intervals, and 3–5 min of active breaks between sets (heart rate should not drop too much during the active breaks) [9,10,11,14]. Exercise intensity should be 85–95% of maximum heart rate (HR_max_) during sets (individuals should breathe heavily and be unable to speak in full sentences) [9,10,11,14]. Exercises can be switched after each interval, but can also kept the same for the whole set/session. As an alternative to using time as a reference, the number of repetitions may be used (10–20 repetitions per interval) [15]. The training regime should be performed three times per week [14].

Based on the presented literature [9,10,11,12,13,14] and considering the current circumstances, we suggest an exercise regime performed as I-HIIT, including bodyweight exercises involving a large amount of muscle mass to induce adaptations in both the cardiorespiratory system and the skeletal musculature. Suitable exercises include jogging in place, burpees, rope skipping, mountain climbers, air-squats, jumping lunges, push-ups, and single-step climbing [11]. The choice of exercises should take the individual’s physical condition into account. After an adequate 10 min, full-body warm-up at 60–70% of HR_max_ [11,14], the training could proceed as illustrated in Figure 1. As explained in Chapter 4, exercise-naïve individuals and those with pre-existing health problems may need to start exercising at lower intensity.

## 4. Safety

HIIT, performed in the suggested intensity zone, has previously been shown to be safe in healthy individuals as well as in patients with hypokinetic diseases [9,11,14]. However, for high-risk patients, relevant contraindications (e.g., patients with unstable angina pectoris, uncompensated heart failure, recent myocardial infarction within the last four weeks, uncontrolled diabetes mellitus, hypertension > 180/110 mmHg, or severe neuropathy) need to be taken into consideration [14]. Furthermore, exercise-naïve individuals and people with preexisting health problems should confer with their general practitioner or an exercise professional before starting this training regime. Initial supervision is recommended for individuals with little experience in exercise training. Consequently, if exercises and intensities adequate to the individual’s physical condition are selected and no contraindications are present, this type of training may be safe for the vast majority of the general population.

## 5. Conclusions

In conclusion, in the current situation initiated by the COVID-19 pandemic, where physical activity levels in the general population have dropped markedly below those commonly seen, it is of major importance to maintain or improve physical fitness, and consequently prevent an exacerbation of the risk for hypokinetic diseases and all-cause mortality. The present evidence-based exercise recommendations are a safe and effective strategy to achieve these goals in healthy individuals and persons with pre-existing medical conditions, provided there are no medical contraindications. These might have important implications for public health during and also after the present pandemic.
